# P-882. Oral Antibiotic Stepdown Therapy for Uncomplicated Enterococcus Blood Stream Infections: A Retrospective Cohort Study

**DOI:** 10.1093/ofid/ofaf695.1090

**Published:** 2026-01-11

**Authors:** Cristina J Torres, Evangeline Green, Elizabeth Lyden, Jasmine R Marcelin, Mackenzie R Keintz

**Affiliations:** University of Nebraska Medical Center, Omaha, Nebraska; University of Nebraska Medical Center, Omaha, Nebraska; University of Nebraska Medical Center, Omaha, Nebraska; University of Nebraska Medical Center, Omaha, Nebraska; University of Nebraska Medical Center, Omaha, Nebraska

## Abstract

**Background:**

Despite increasing evidence supporting oral antibiotic therapy (OAT) for uncomplicated bloodstream infections (uBSI), data specific to Enterococcus bloodstream infections are limited. Therefore, we evaluated clinical outcomes associated with OAT versus continued intravenous (IV) therapy for Enterococcus uBSI.

**Figure:**
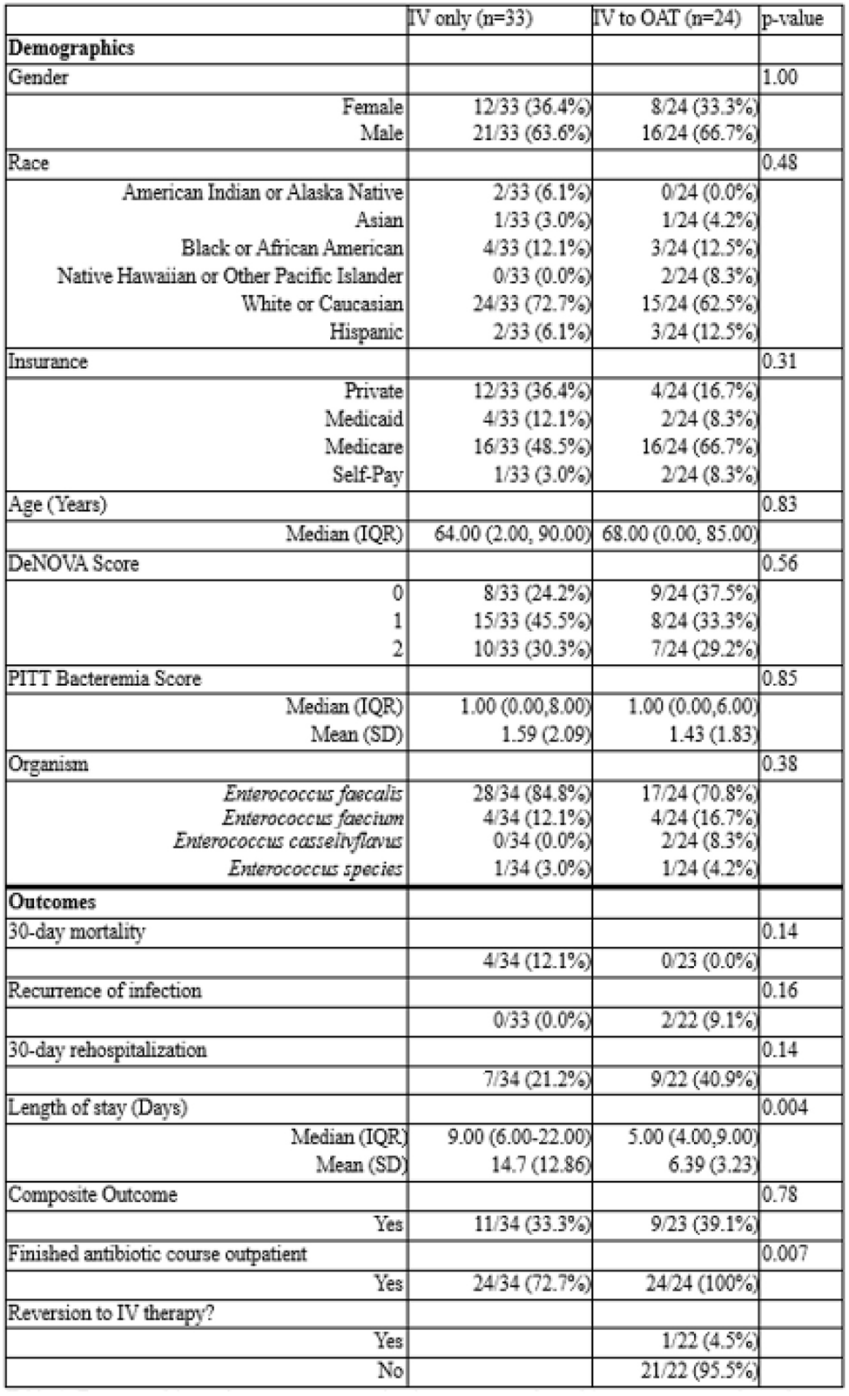
Demographics and outcomes comparing intravenous only and intravenous to oral stepdown therapy IV= intravenous, OAT= oral antimicrobial therapy, IQR= interquartile range, SD= Standard Deviation

**Figure:**
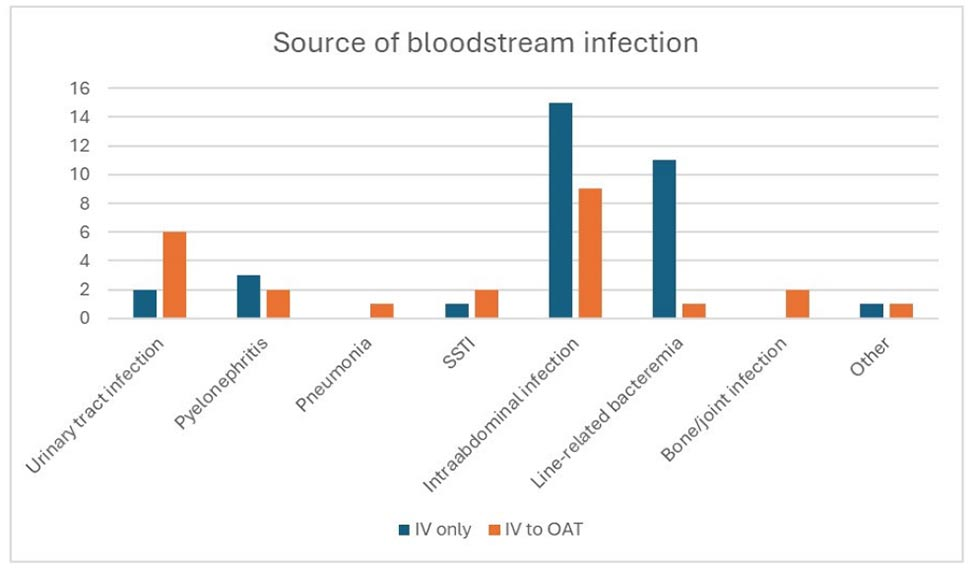
IV= intravenous, OAT= oral antimicrobial therapy, SSTI= skin and soft tissue infection

**Methods:**

We conducted a retrospective cohort study of hospitalized adults diagnosed with uBSI due to Enterococcus species between 1/1/2013, and 12/31/2019. Patients were excluded if they had endovascular, central nervous system, or uncontrolled bone/joint infections; lacked documented blood culture clearance; or remained febrile after 72 hours of effective therapy. We compared outcomes between patients who received only IV therapy and those transitioned to OAT. Primary outcomes included hospital length of stay (LOS), 30-day readmission, 30-day mortality, and adverse drug events. A composite outcome comprising 30-day mortality, 30-day hospital readmission, and infection recurrence was used. Fisher’s exact test, t-tests, and Wilcoxon rank sum tests were used for comparisons.

**Figure:**
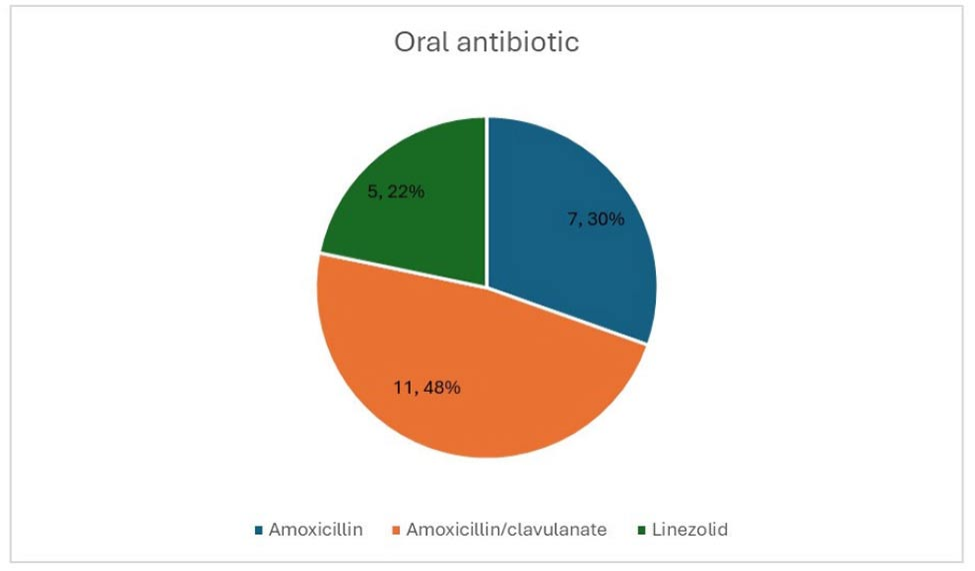
Choice of stepdown oral antibiotic in IV to OAT group IV= intravenous, OAT= oral antimicrobial therapy

**Results:**

Of 493 Enterococcus BSI cases identified, 57 met inclusion criteria for uBSI. Baseline demographics and Pitt bacteremia scores were similar between groups (Table 1). Patients receiving OAT (n=24) were more likely to have a urinary source and less likely to have intra-abdominal or central line–associated sources (p< 0.05) (Figure 1). Amoxicillin-clavulanate was the most used agent for OAT (Figure 2). OAT was associated with a shorter median hospital LOS (5 vs. 9 days, p< 0.05) and higher rates of outpatient antibiotic completion (p< 0.05). In the IV group (n=33), 30-day mortality occurred in 4 patients versus none in the OAT group (p=0.14). Infection recurrence occurred in 2 OAT patients and none in the IV group (p=0.16). There were no significant differences in the composite adverse outcome (39.1% OAT vs. 33.3% IV, p=0.78) or 30-day readmission rates (40.9% OAT vs. 21.2% IV, p=0.14).

**Conclusion:**

Oral stepdown therapy for uBSI was associated with reduced hospital LOS without increased adverse outcomes, highlighting potential opportunities for antimicrobial stewardship in the management of Enterococcus bloodstream infections.

**Disclosures:**

All Authors: No reported disclosures

